# Integrated evaluation of plant oils, entomopathogenic fungi and insecticides against rugose spiralling whitefly (*Aleurodicus rugioperculatus* Martin) on coconut

**DOI:** 10.3389/finsc.2026.1829366

**Published:** 2026-06-09

**Authors:** Priyanka Borbaruah, Inee Gogoi, Samiron Pathak, Badal Bhattacharyya, Shimantini Borkataki, K. Sindhura Bhairavi, Bandana Saikia, Jabanika Hazarika, Partha Pratim Gyanudoy Das, Mousumi Phukon, Nang Sena Manpoong, Elangbam Bidyarani Devi

**Affiliations:** 1 Department of Entomology, AAU-Citrus and Plantation Crop Research Institute, Tinsukia, Assam, India; 2 Department of Entomology, Assam Agricultural University, Jorhat, Assam, India; 3 Department of Entomology, AAU-Horticulture Research Station, Kahikuchi, Assam, India; 4 Department of Plant Pathology, Sarat Chandra Sinha College of Agriculture, Dhubri, Assam, India

**Keywords:** Acetamiprid, *Aleurodicus rugioperculatus*, coconut, integrated pest management, Lecanicillium lecanii, plant oils

## Abstract

The rugose spiralling whitefly (RSW), *Aleurodicus rugioperculatus* Martin (Hemiptera: Aleyrodidae), has emerged as a serious invasive pest of coconut in India since its first report in 2016. Its rapid population build-up, coupled with profuse honeydew excretion and sooty mould development, causes substantial reduction in photosynthesis and nut yield. The present study aimed to develop and validate IPM modules by combining mechanical, botanical, microbial and need-based chemical tactics on the basis of laboratory screening and field evaluation. In laboratory bioassays, castor oil, *Lecanicillium lecanii* and acetamiprid caused the highest mortality and thus were incorporated in Module 3, which was further compared against two institutionally derived modules for field validation. Significant differences among the treatments were observed (*p* < 0.05), Across two seasons, all IPM modules reduced rugose spiralling whitefly infestation relative to the untreated control, but Module 1 achieved the greatest overall pest suppression (>70%) and the highest yield increase (28.4%). However, Module 3, integrating yellow sticky traps, 1% starch wash, castor oil, *L. lecanii* and acetamiprid, provided substantial suppression with lower dependence on conventional chemical input. On the basis of efficacy, ecological compatibility and field practicability, Module 3 is recommended as the preferred IPM option for routine management of *A. rugioperculatus* in coconut under north-eastern Indian conditions, whereas Module 1 may be reserved for severe outbreak situations requiring rapid suppression. The study demonstrates that laboratory-validated botanical, microbial and selective chemical components can be assembled into a scalable and recommendation-driven IPM programme for sustainable management of rugose spiralling whitefly.

## 1 Introduction

The rugose spiraling whitefly (RSW), *Aleurodicus rugioperculatus* Martin (Hemiptera: Aleyrodidae), is a highly invasive pest of coconut and other perennial crops in Asia. Native to Central America, the pest was first reported in India in 2016 (1), and since then it has spread rapidly across the coconut-growing regions of Southern and North-Eastern India (2, 3). The insect colonizes the abaxial surface of coconut leaves, producing copious honeydew that promotes the growth of sooty mould fungi. Heavy infestations lead to impaired photosynthesis, reduced nut yield, and weakening of palms. Its polyphagy and high fecundity have facilitated its rapid establishment in diverse agroclimatic zones.

Management of RSW has largely relied on chemical insecticides, particularly neonicotinoids such as imidacloprid and thiamethoxam (4). While effective in the short term, these chemicals raise concerns regarding resistance development, non-target impacts, environmental contamination, and disruption of natural enemies. Repeated use of neonicotinoids may lead to resistance development through mechanisms such as enhanced detoxification and target-site modification, as observed in whiteflies such as *Bemisia tabaci* (5). Moreover, the thick waxy layer covering RSW nymphs reduces the efficacy of contact insecticides (6). Hence, reliance on insecticides alone is unsustainable. Plant oils and entomopathogenic fungi (EPF) represent promising alternatives. Plant oils such as neem, pongamia, and castor are known for their antifeedant, ovicidal, and repellent properties, and have demonstrated efficacy against various sucking pests including whiteflies and mites (7–9). EPF such as *Lecanicillium lecanii* and *Beauveria bassiana* have been successfully employed against whiteflies (*Bemisia tabaci* and *Aleurodicus dispersus*) under laboratory and field conditions (10, 11). Assam’s humid and warm climate is also favourable for EPF persistence and infection, enhancing their effectiveness under field conditions. Additionally, natural enemies such as *Encarsia* spp. play a crucial role in regulating whitefly populations, underscoring the need for integrated approaches.

Recent studies demonstrate that plant oils, entomopathogenic fungi (EPF), and insecticides are often studied either individually or in partial combinations. Integration of plant extracts with EPF has been shown to enhance pest suppression compared to individual applications (12), but most of these studies typically do not include simultaneous comparison with synthetic insecticides under an experimental framework. To date, studies seldom compare botanicals, EPF, and insecticides together against both adults and nymphs of this whitefly, nor validate such findings through well-replicated field trials on coconut. Institutional recommendations from ICAR-CPCRI and ICAR-NBAIR include individual components like neem oil sprays, *Encarsia* releases, or EPF applications, but these have not been integrated based on laboratory efficacy into robust IPM modules. However, studies on the comparative efficacy of these eco-friendly agents against RSW remain limited, particularly under northeastern Indian conditions. Despite the availability of individual management components recommended by various ICAR agencies, there is limited evidence on their comparative efficacy under uniform experimental conditions and lack of integration based on laboratory-validated performance, particularly under northeastern Indian agroecosystems. Given the invasive nature of RSW and the limitations of conventional control measures, there is an urgent need to evaluate alternative strategies and to integrate them into an ecologically sustainable pest management module. Integrated application of plant oils, EPF, and selective insecticides could result in significantly greater suppression of *A. rugioperculatus* compared to individual or partially integrated approaches.

The present study was therefore undertaken to (i) evaluate mortality and LC_50_ values of plant oils, entomopathogenic fungi, and systemic neonicotinoid insecticides (acetylcholine receptor agonists; IRAC Group 4A) such as acetamiprid, imidacloprid, and thiamethoxam, along with lipid biosynthesis inhibitors (tetronic acid derivatives; IRAC Group 23) such as spiromesifen against RSW under laboratory conditions, and (ii) assess their impact on pest reduction, yield improvement, and economic returns in field experiments to develop an integrated pest management (IPM) module tailored for coconut ecosystems in Assam, India.

## 2 Materials and methods

### 2.1 Study sites and test insect

Laboratory experiments were conducted in the Post Graduate Laboratory, Department of Entomology, Assam Agricultural University (AAU), Jorhat, Assam, India (26.72°N, 94.19°E). Adults of RSW were collected from infested coconut palms (*Cocos nucifera* cv. Kamrupa) at the Horticultural Experimental Farm, AAU, Jorhat. Collected leaves were examined under a stereo zoom microscope to remove parasitized individuals of *Encarsia* spp. prior to use in bioassays. RSW were identified, and both adults and nymphs were collected using aspirators and leaf cuttings for laboratory bioassays. Insects were maintained in aerated cages under controlled laboratory conditions (27 ± 2 °C; 70 ± 5% RH; 12:12 h L:D photoperiod) until experimentation. For laboratory bioassays, individuals of similar developmental stage and size were selected to ensure uniformity. In field experiments, experimental units were selected based on comparable initial infestation levels to minimize variability among treatments.

### 2.2 Laboratory bioassays

#### 2.2.1 Plant oils

Three plant oils *viz*., jatropha (*Jatropha curcas*), karanj (*Pongamia pinnata*), and castor (*Ricinus communis*), were evaluated along with neem oil (*Azadirachta indica*) as the standard check. Stock solutions (10%) were prepared in distilled water with 0.1% emulsifier (soap solution). Serial dilutions yielded working concentrations of 0.2, 0.5, 1, 2, and 3%. Bioassays were conducted by direct spray method (3, 13) with modifications. Adults and late-instar nymphs were confined on coconut leaf discs placed in Petri dishes (9 cm diameter) and test solutions were applied uniformly using an atomizer until incipient runoff. Spray applications were standardized by delivering approximately 1.5–2 ml solution per Petri dish, producing fine droplets (approximately 100–150 µm) to ensure uniform coverage. Each treatment included five concentrations plus control, replicated three times with 20 insects per replicate. Mortality was assessed at: 24, 48, and 72 h after treatment (HAT). Control treatments consisted of distilled water with emulsifier. Environmental conditions (27 ± 2 °C and 70 ± 5% RH) were monitored and maintained uniformly throughout the experiments.

#### 2.2.2 Entomopathogenic fungi

Isolates of *B. bassiana*, *Metarhizium anisopliae*, *L. lecanii* and *Isaria fumosorosea* were procured from ICAR–NBAIM, Uttar Pradesh. *I. fumosorosea* was used as the standard check, showing approximately 85% mortality under laboratory conditions. The fungal isolates were selected based on prior laboratory maintenance and preliminary screening for pathogenicity. The EPF cultures were maintained in Potato dextrose agar (PDA; HiMedia) prepared at 39 g/L in double-distilled water, adjusted to pH 5.6, and sterilized at 121 °C for 15 min. Media were amended with streptomycin sulfate (0.5 g/L) and poured aseptically into 90-mm Petri plates. Fungal isolates were subcultured under laminar airflow and incubated at 25 ± 1 °C for 14 days. To prepare the spore suspension, conidia were harvested from sporulating plates by gently scraping the colony surface and suspending spores in sterile distilled water with 0.02% Tween-80. Initial stock suspensions were quantified using a Neubauer hemocytometer and adjusted by serial dilution to 10³, 10^5^, 10^7^, 10^8^, and 10^9^ conidia/ml. Prior to bioassays, spore viability was assessed using germination tests, and only suspensions exhibiting ≥80% germination were used to ensure uniform infectivity across isolates. For each EPF isolate, 0.05 ml of conidial suspension was spread on agar-coated slides (five replications per treatment) and incubated at 26 ± 1 °C for 24 h. Germination percentage was recorded under 400× magnification, following Francisco et al. (14). Coconut leaf discs infested with adults or nymphs were dipped in the suspensions for 30 s, air-dried, and maintained in Petri plates lined with moist filter paper. Mortality was recorded at 3, 5, and 7 days after treatment (DAT). Further, dead insects were surface-sterilized and incubated to confirm mycosis.

#### 2.2.3 Insecticides

To strengthen resistance-management interpretation, the tested insecticides were classified according to IRAC mode of action: acetamiprid, imidacloprid and thiamethoxam belong to Group 4A (nicotinic acetylcholine receptor competitive modulators; neonicotinoids), whereas spiromesifen belongs to Group 23 (inhibitors of acetyl-CoA carboxylase; tetronic acid derivative). The treatments constituted Thiamethoxam 25% WG, Spiromesifen 22.9% SC, and Acetamiprid 20% SP were tested, with Imidacloprid 17.8% SL as the standard check. Stock solutions (1%) were prepared by dissolving the required quantity in distilled water, followed by serial dilution to obtain 0.001–1% concentrations. Treatments were done similar to plant oils and mortality was assessed at: 24, 48, and 72 h after treatment (HAT).

### 2.3 Field experiment

Field experiment was carried out at AAU- Horticulture Research Station, Kahikuchi, Kamrup, Assam, India (20°18 N and 91°78 E). The experiment was carried out on 10 years old coconut palms in two seasons, viz., June-August, 2022 and June-August, 2023, when the pest intensity was high. Each experimental unit consisted of a single palm spaced at 7.5 × 7.5 m. Adequate buffer spacing was maintained between treatments to avoid spray drift.

#### 2.3.1 Layout and design

The treatment combinations were selected to compare two institutionally recommended modules with one experimental module developed from laboratory efficacy screening. Mechanical and canopy-cleaning tactics (yellow sticky traps and 1% starch wash) were maintained as common baseline measures to reduce adult load and sooty mould across all active modules. Module 1 represented an adapted high-suppression benchmark integrating trap-based monitoring, botanical suppression, parasitoid release, scavenger conservation and a curative insecticidal component. Module 2 represented a biological-control-led benchmark centred on *Encarsia* spp. and *Isaria fumosorosea*. Module 3 was the laboratory-validated reduced-chemical module, in which castor oil, *Lecanicillium lecanii* and acetamiprid were selected because they were the most effective or statistically comparable top-performing agents within their respective groups under laboratory conditions. The experiment was laid out in randomized block design with four modules and six replications (one palm per replication). Each palm received treatments according to the respective module (**Table 1**).

**Table 1:** Details of the IPM modules evaluated in the experiment.

Modules	Treatments
Module 1*	Installation of yellow sticky trap+ Spraying of 1% starch solution+ Spraying of neem oil 0.5%+ Release of parasitoid *Encarsia* spp.*+* Release of sooty mould feeding scavenging beetle *Leiochrinus nilgirianus +* Spraying of Imidacloprid 0.005%.
Module 2**	Installation of yellow sticky trap + Spraying of 1% starch solution + Release of parasitoid *Encarsia* spp. + Spraying of EPF *Isaria fumosorosea*
Module 3	Installation of yellow sticky trap + Spraying of 1% starch solution +Spraying of castor oil + Spraying of spore suspension of EPF *Lecanicillium lecanii* + Spraying of Acetamiprid 20% SP
Module 4	Untreated control

*Module 1: Recommended by ICAR- Central Plantation Crops Research Institute (CPCRI), Kasaragod, Kerala.

**Module 2: Recommended by ICAR- National Bureau of Agricultural Insect Resource *(NBAIR), Bengaluru, Karnataka.*

Sprays were applied with a foot sprayer, using 5–10 L solution per palm depending on canopy size. Parasitoid release was performed by stapling leaf bits containing parasitized puparia onto fronds. From each palm, four fronds (one per direction) were selected, and five leaflets per frond were examined. Pre-treatment counts were recorded one day prior to first spray, and post-treatment observations were taken at 85 days after initial application. Parameters included: pest incidence (% infested leaflets), damage intensity (% leaf area covered with sooty mould), number of adults per leaflet, number of egg spirals per leaflet.

### 2.4 Statistical analysis

All laboratory bioassays were analyzed under a completely randomized design (CRD) with three independent replicates per treatment–concentration–time combination (20 insects per replicate). The sample size (n = 3 replicates with 20 insects each) was based on standard bioassay protocols and was sufficient to detect statistically significant differences among treatments. Mortality data (per cent) were angular transformed using the arcsine square-root transformation prior to analysis to stabilise variances; back-transformed means are reported for ease of interpretation where relevant. One-way ANOVA was performed separately for each product class (plant oils, entomopathogenic fungi, and insecticides) across the five concentrations (or spore dilutions) at each observation time (plant oils and insecticides at 24, 48, and 72 HAT; fungi at 3, 5, and 7 DAT). Treatment means were separated using the critical difference at α = 0.05 (CD, P = 0.05). Median lethal concentrations (LC_50_) and their 95% fiducial limits were estimated by probit regression (Finney’s method), using log_10_ concentration as the predictor; model fit was evaluated with χ² goodness-of-fit and the standard error of the slope. Probit analyses were conducted in SPSS (v. 12.0). Conidial density and spore germination data for the fungal pathogens were analysed by one-way ANOVA across serial dilutions with the same mean separation criterion (CD, P = 0.05); percentage germination was analyzed on angular-transformed values.

Field experiments were analyzed as a randomized block design (RBD) with six blocks (replications) and four IPM modules as fixed treatments, conducted over two seasons (June–August 2022 and June–August 2023). Pre-treatment counts were taken 1 day before imposing treatments, and post-treatment assessments were made 85 days after the first intervention. For incidence and damage intensity (percent), data were angular transformed before analysis; count variables (number of adults per leaflet and number of egg spirals per leaflet) were analyzed after square-root transformation [√(x + 0.5)] when required by residual diagnostics. A two-way ANOVA with factors Module and Season and Block (Season) as the random term was fitted for each response; when the Module × Season interaction was non-significant, main-effect means were interpreted; otherwise, simple effects were examined within seasons. Mean separation followed the CD (P = 0.05) procedure used throughout. Figures are presented for visualization of treatment trends, while statistical significance is interpreted based on ANOVA and mean separation tests presented in tables.

## 3 Results

### 3.1 Laboratory evaluation of plant oils

**Figure 1:**
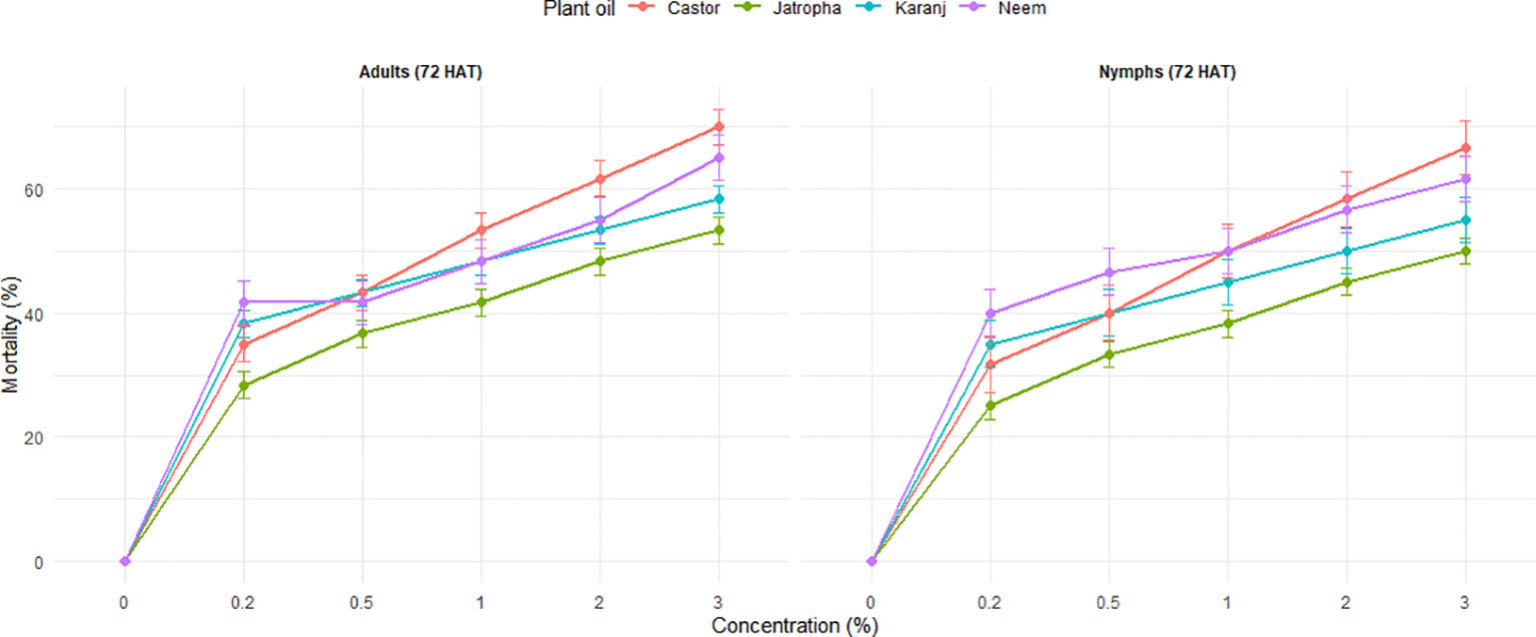
Mortality of *Aleurodicus rugioperculatus* adults vs. nymphs under plant oil treatments.

Bioassays with different plant oils revealed significant variation in adult and nymphal mortality (**Supplementary Tables 1** & **2**). Among the oils tested, castor oil exhibited the highest efficacy, causing up to 70.0% adult mortality and 66.7% nymphal mortality at 3% concentration after 72 h of exposure. Neem oil, used as the standard check, recorded 65.0% and 61.7% mortality at 3% concentration against adults and nymphs, respectively, at 72 h. Karanj oil was moderately effective (58.3% adult and 55.0% nymphal mortality), whereas jatropha oil was the least effective (53.3% and 50.0% mortality, respectively, at 3% concentration after 72 h) (**Figure 1**).

Probit analysis indicated LC_50_ values ranging from 0.73% (castor oil) to 2.25% (jatropha oil) for adults, and from 0.96% (castor oil) to 2.76% (jatropha oil) for nymphs. The overall order of toxicity at 72 h was: castor oil > neem oil > karanj oil > jatropha oil (**Table 2**).

**Table 2:** LC_50_ values of plant oils against adults and nymphs of *Aleurodicus rugioperculatus* at 72 HAT.

Treatment	Regression equation	Standard error of regression coefficient	Chi square	LC_50_ (per cent)	Fiducial limit		Order of toxicity
					Lower limit	Upper limit	
Adults							
Jatropha oil	Y= -1.92 + 0.54X	0.08	3.75	2.25	1.64	3.62	IV
Karanj oil	Y= -0.02 + 0.42X	0.08	3.88	1.14	0.80	1.73	III
Castor oil	Y= 0.10 + 0.76X	0.08	8.16	0.73	0.59	0.89	I
Neem seed oil (standard check)	Y= 0.13 + 0.45X	0.08	13.91	0.86	0.62	1.18	II
Nymphs							
Jatropha oil	Y= -0.58 + 0.21X	0.03	8.92	2.76	2.34	3.44	IV
Karanj oil	Y= -0.35 + 0.15X	0.03	15.12	2.24	1.80	3.00	III
Castor oil	Y= -0.29 + 0.30X	0.03	20.84	0.96	0.60	1.25	I
Neem seed oil (standard check)	Y= -0.21 + 0.18X	0.03	12.10	1.19	0.80	1.55	II

*Mean of three replicates (95% fiducial limits shown Y = Probit kill, X = log concentration).

### 3.2 Pathogenicity of entomopathogenic fungi

**Figure 2:**
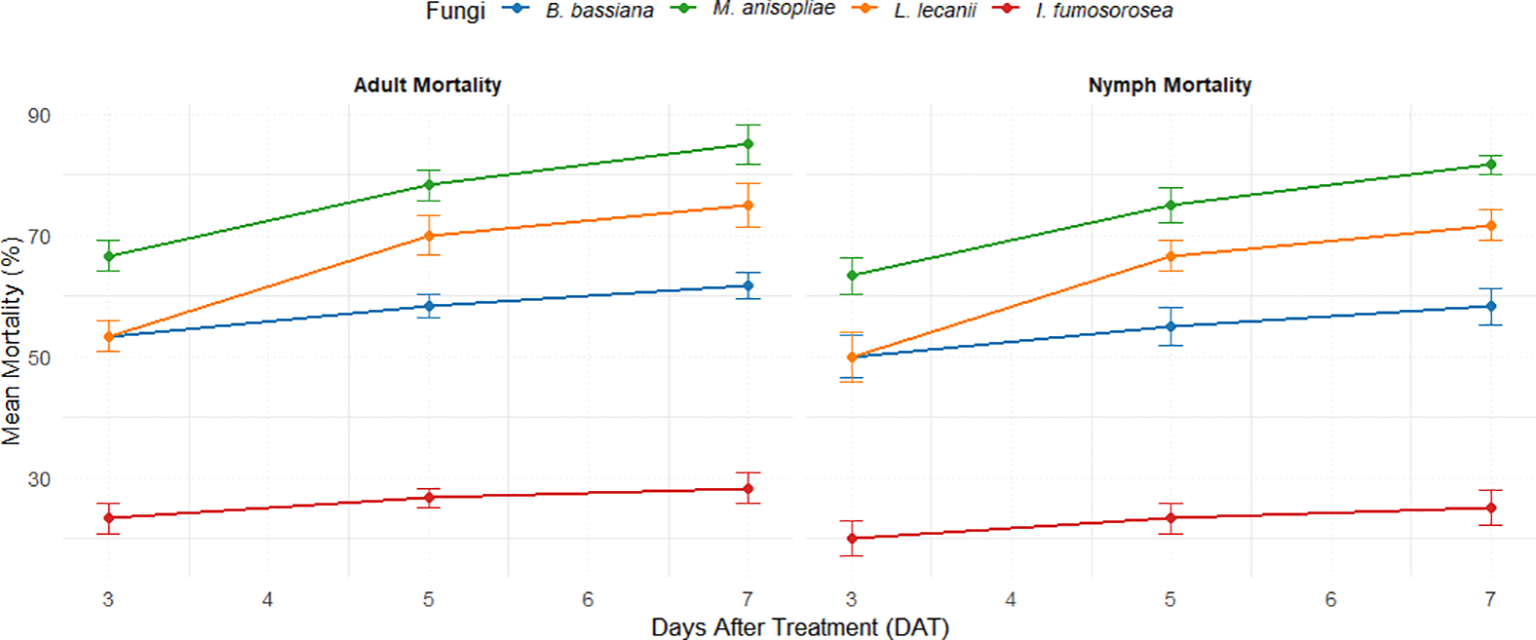
Effect of entomopathogenic fungi (10^7^ spores/ml) on *A. rugioperculatus* mortality.

All tested entomopathogenic fungi (EPF) were pathogenic to *A. rugioperculatus* adults and nymphs (**Supplementary Tables 3** & **4**). *L. lecanii* showed the highest mortality among the non-standard fungi, with 75.0% adult and 71.7% nymphal mortality at 10^7^ conidia mL⁻¹ after 7 days. This was followed by *B. bassiana*, which caused 61.7% and 58.3% mortality in adults and nymphs, respectively. *Metarhizium anisopliae* showed comparatively lower pathogenicity (<30% mortality at 7 DAT). The standard check, *I. fumosorosea*, was most effective overall, inducing 85.0% mortality in adults and 81.7% in nymphs at 10^7^ conidia mL⁻¹ after 7 DAT (**Figure 2**).

Conidial density and germination assays confirmed the biological superiority of *L. lecanii* and *I. fumosorosea*. *L. lecanii* produced the highest viable propagule density (12.74 × 10^7^ conidia mL⁻¹) with 84.0% germination, while *I. fumosorosea* recorded the highest overall sporulation rate (87.5% at 10^7^ dilution). *M. anisopliae* showed the lowest viability and pathogenicity parameters (**Table 3**).

**Table 3:** Effect of entomopathogenic fungi on mortality of *A. rugioperculatus*.

Treatment (fungus, 10^7^ conidia mL⁻¹)	Adult mortality (%)	Nymph mortality (%)	Sporulation (%)	Conidial density (×10^7^ mL⁻¹)	Germination (%)	Rank
*Isaria fumosorosea*	85.0 ± SE a	81.7 ± SE a	87.5	–	–	I
*Lecanicillium lecanii*	75.0 ± SE b	71.7 ± SE b	82.0	12.74	84.0	II
*Beauveria bassiana*	61.7 ± SE c	58.3 ± SE c	78.0	9.12	79.5	III
*Metarhizium anisopliae*	28.3 ± SE d	25.0 ± SE d	65.0	5.38	70.2	IV

*Different letters indicate significant differences (*P* < 0.05) (n=3).

### 3.3 Effect of insecticides on *Aleurodicus rugioperculatus*

Marked differences in adult mortality were observed among the insecticides tested (**Table 4**). Among the treatments, imidacloprid 17.8% SL recorded the highest adult and nymphal mortality (95.0% and 93.3%, respectively), followed by acetamiprid 20% SP (91.7% and 90.0%). However, both insecticides were statistically at par, as indicated by the same significance grouping. Thiamethoxam 25% WG showed comparatively lower efficacy, with 88.3% adult and 85.0% nymphal mortality, and was significantly different from imidacloprid and acetamiprid. Spiromesifen 22.9% SC recorded the lowest mortality (80.0% in adults and 78.3% in nymphs), and was significantly inferior to all other treatments. Overall, imidacloprid showed the greatest intrinsic toxicity under laboratory conditions, as reflected by the lowest LC_50_ values and the highest nominal mortality; however, its 72 HAT mortality was statistically at par with acetamiprid, indicating that both compounds were comparably effective within the tested concentration range.

**Table 4:** Mortality of *A. rugioperculatus* adults and nymphs due to insecticide treatments at 72 HAT.

Insecticide	Adult mortality (%)	Nymph mortality (%)	Rank
Imidacloprid 17.8% SL	95.0 ± SE a	93.3 ± SE a	I
Acetamiprid 20% SP	91.7 ± SE a	90.0 ± SE a	II
Thiamethoxam 25% WG	88.3 ± SE b	85.0 ± SE b	III
Spiromesifen 22.9% SC	80.0 ± SE c	78.3 ± SE c	IV

*Means followed by different letters differ significantly (*P* < 0.05) (n = 3).

### 3.4 Toxicity of plant oils and chemical insecticides against *Aleurodicus rugioperculatus*

Probit analysis revealed clear differences in the susceptibility of adults and nymphs of *A. rugioperculatus* to both plant oils and synthetic insecticides with statistically significant variations (P < 0.05) (**Table 5**). Among plant oils, adults were slightly more susceptible than nymphs: castor oil showed the lowest LC_50_ in adults (0.73%; 95% CI: 0.59–0.89) compared with 0.96% (95% CI: 0.60–1.25) in nymphs, followed by neem (0.86%; 95% CI: 0.62–1.18), karanj (1.14%; 95% CI: 0.08–1.73), and jatropha (2.25%; 95% CI: 1.64–3.62). In contrast, synthetic insecticides demonstrated much higher potency, again with adults generally more sensitive than nymphs. Imidacloprid recorded extremely low LC_50_ values in both stages (0.006% in adults; 95% CI: 0.004–0.007 and 0.01% in nymphs; 95% CI: 0.008–0.03), followed by acetamiprid (0.07% in adults; 95% CI: 0.06–0.09 and 0.07% in nymphs; 95% CI: 0.06–0.15), thiamethoxam (0.32% in adults; 95% CI: 0.26–0.39 and 0.58% in nymphs; 95% CI: 0.48–0.72), and spiromesifen (0.89% in adults; 95% CI: 0.67–1.34 and 0.94% in nymphs; 95% CI: 0.86–1.04). Slope and χ² statistics indicated satisfactory model fit across treatments. Overall, while the rank order remained consistent (castor > neem > karanj > jatropha for plant oils; imidacloprid > acetamiprid > thiamethoxam > spiromesifen for insecticides), the consistently lower LC_50_ values in adults highlight their relatively higher susceptibility compared with nymphs.

**Table 5:** Comparative LC_50_ values of plant oils and insecticides against *A. rugioperculatus* at 72 HAT.

Treatment (oil)	Life stage	Regression equation (probit = f(log conc.))	Std. error (reg. coeff.)	χ²	LC_50_ (%)	95% fiducial limits (%)	Toxicity rank
Jatropha oil	Adult	Y = −1.92 + 0.54 X	0.08	3.75	2.25	1.64 – 3.62	IV
Karanj oil	Adult	Y = −0.02 + 0.42 X	0.08	3.88	1.14	0.80 – 1.73	III
Castor oil	Adult	Y = 0.10 + 0.76 X	0.08	8.16	0.73	0.59 – 0.89	I
Neem seed oil	Adult	Y = 0.13 + 0.45 X	0.08	13.91	0.86	0.62 – 1.18	II
Jatropha oil	Nymph	Y = −0.58 + 0.21 X	0.03	8.92	2.76	2.34 – 3.44	IV
Karanj oil	Nymph	Y = −0.35 + 0.15 X	0.03	15.12	2.24	1.80 – 3.00	III
Castor oil	Nymph	Y = −0.29 + 0.30 X	0.03	20.84	0.96	0.60 – 1.25	I
Neem seed oil	Nymph	Y = −0.21 + 0.18 X	0.03	12.10	1.19	0.80 – 1.55	II
Imidacloprid	Adult	Y = 1.52 + 0.67X	0.67 ± 0.06	12.90	0.006	0.004–0.007	I
Acetamiprid	Adult	Y = 1.05 + 0.95X	0.95 ± 0.08	10.47	0.07	0.06–0.09	II
Thiamethoxam	Adult	Y = 0.39 + 0.80X	0.80 ± 0.07	9.94	0.32	0.26–0.39	III
Spiromesifen	Adult	Y = 0.03 + 0.78X	0.78 ± 0.09	22.70	0.89	0.67–1.34	IV
Imidacloprid	Nymph	Y = −0.29 + 1.70X	1.70 ± 1.84	67.17	0.01	0.008–0.03	I
Acetamiprid	Nymph	Y = −0.07 + 1.19X	1.19 ± 0.10	21.42	0.07	0.06–0.15	II
Thiamethoxam	Nymph	Y = −0.59 + 1.00X	1.00 ± 0.09	22.47	0.58	0.48–0.72	III
Spiromesifen	Nymph	Y = −0.92 + 0.98X	0.98 ± 0.10	7.57	0.94	0.86–1.04	IV

*Mean of three replicates (95% fiducial limits shown Y = Probit kill, X = log concentration).

### 3.5 Field evaluation of IPM modules

Field trials demonstrated that integrated pest management (IPM) modules varied significantly in their ability to suppress *A. rugioperculatus* populations under natural conditions. Among the evaluated treatments, Module 1, which integrated chemical, plant oils, and biological components, consistently recorded the lowest whitefly incidence, with mean adult and nymphal populations reduced by >70% compared with the untreated control. This superior performance may be attributed to the combined action of insecticides, plant oils, and biological control agents, including parasitoids, which together enhanced pest suppression. Module 2 was the next most effective, providing >60% reduction in pest populations, while Module 3, comprising castor oil, *L. lecanii*, and acetamiprid along with mechanical measures, also resulted in substantial suppression, though comparatively lower than Module 1. Cost-benefit analysis indicated that although chemical-only modules had slightly higher immediate pest knockdown, IPM modules integrating plant oils and fungi achieved better sustainability indices, highlighting their potential for adoption in farmer fields. Higher yield was observed in treatments that recorded greater reduction in adult and nymphal populations, indicating that effective pest suppression contributed to improved productivity under field conditions.

### 3.6 Comparative effectiveness of IPM modules

Pooled analysis over two seasons showed significant variation in the efficacy of IPM modules (P < 0.05) against *A. rugioperculatus* (**Table 6**). Module 1 recorded the greatest suppression of both adults (73.5 ± 2.1%) and nymphs (72.0 ± 2.0%), with a 28.4% increase in yield and the highest benefit:cost ratio (1:4.6). Module 2 was the next best (64.0 ± 2.5% adult and 62.3 ± 2.2% nymph reduction; 23.9% yield increase; B:C ratio 1:4.1). In contrast, Module 3 and Module 4 offered only moderate suppression (35–45%) with modest yield gains (13–16%). The untreated control maintained the highest pest pressure and lowest yield. Overall, the results confirmed the superiority of Module 1, integrating chemical, plant oils, and biological interventions, as a sustainable and economically viable approach for whitefly management across seasons.

**Table 6:** Effect of IPM modules on population reduction and yield of *A. rugioperculatus* over two seasons.

Module	Adult reduction (%)	Nymph reduction (%)	Mean yield (t/ha)	Yield increase over control (%)	Benefit:cost ratio	Rank
Module 1	73.5 ± 2.1	72.0 ± 2.0	2.85 ± 0.08	28.4	1:4.6	I
Module 2	64.0 ± 2.5	62.3 ± 2.2	2.68 ± 0.06	23.9	1:4.1	II
Module 3	42.1 ± 2.8	40.6 ± 2.6	2.35 ± 0.07	15.6	1:3.2	III
Module 4	37.8 ± 2.9	35.4 ± 2.7	2.26 ± 0.05	13.2	1:2.9	IV

*Data represents mean of six replicates (n=6).

## 4 Discussion

The present investigation demonstrated that plant oils, entomopathogenic fungi (EPF), and chemical insecticides varied considerably in their efficacy against both adult and nymphal stages of *A. rugioperculatus* (rugose spiraling whitefly, RSW). Among the plant oils tested, castor oil consistently exhibited higher efficacy than other oils. These results align with earlier reports highlighting the ovicidal, repellent, and toxic properties of neem formulations against whiteflies and other sucking pests (3, 15, 16). Castor oil’s effectiveness can be attributed to ricinoleic acid and its capacity to disrupt cuticular integrity, a mode of action also suggested in earlier evaluations against scale insects and whiteflies (8). Similarly, karanj oil’s bioactivity against sap-feeding insects has been associated with karanjin and related limonoids, though its efficacy is typically lower than neem-based products (7). Botanical extracts like plant oils can significantly reduce insect survival, fecundity, and enzymatic activity, thereby contributing to sustainable pest management (17). Together, these findings confirm the potential of castor and neem oils as botanically derived alternatives for managing RSW within an integrated framework.

Entomopathogenic fungi also showed promising potential, particularly *L. lecanii*, which emerged as a promising isolate, with strong infectivity and sporulation characteristics. The conidial density and sporulation of *L. lecanii* were also superior to the other isolates, although slightly lower than the standard check *I. fumosorosea*. The pathogenicity of *L. lecanii* against whiteflies has been extensively documented; crude extracts and conidial suspensions were shown to induce feeding deterrence and mortality in *B. tabaci* (18). Similarly, *I. fumosorosea* has been reported to infect multiple developmental stages of RSW effectively under both laboratory and field conditions (19). The relative efficacy of *L. lecanii* in the current study reinforces its role as a viable mycoinsecticide against RSW, with additional benefits of safety toward natural enemies and compatibility with plant oils (20).

In laboratory bioassays, imidacloprid exhibited the lowest LC_50_ values and the highest nominal mortality, indicating greater intrinsic toxicity under controlled exposure. However, acetamiprid was statistically at par with imidacloprid at 72 HAT, and the two compounds should therefore be interpreted as comparable in laboratory efficacy rather than contradictory in performance. The reason acetamiprid was retained in Module 3 is not that it exceeded imidacloprid toxicologically, but that module development was based on IPM design criteria in addition to LC_50_ alone, namely the inclusion of one effective chemical component within a reduced-chemical module built around botanical, microbial and mechanical tactics. Thiamethoxam and spiromesifen were comparatively less effective. The order of toxicity observed-Imidacloprid > Acetamiprid > Thiamethoxam > Spiromesifen—is consistent with earlier evaluations of neonicotinoids and tetronic acid derivatives against whiteflies (21, 22). The strong performance of acetamiprid is noteworthy, as its systemic properties and relatively lower resistance issues compared to imidacloprid make it a suitable candidate for inclusion in integrated modules. However, it is to be noted that acetamiprid should be used judiciously within IPM through rotation with different modes of action and integration with plant oils and entomopathogenic fungi, to avoid resistance development in target pests. The interaction between plant-derived compounds and insecticide resistance mechanisms has been increasingly recognized, with studies showing that exposure to phytochemicals can modulate detoxification enzymes such as cytochrome P450, thereby influencing insecticide susceptibility (23, 24).

The findings of the present study align with global efforts to manage invasive whiteflies through integrated pest management strategies that reduce reliance on chemical control. Resistance to neonicotinoids has been widely documented in whitefly species, highlighting the need for diversified approaches that combine chemical, biological, and botanical interventions. In this context, the integration of plant oils, entomopathogenic fungi (EPF), and selective insecticides offers a multi-tactic strategy that can delay resistance development.

From an IPM standpoint, Module 1 should be interpreted as the maximum-suppression benchmark and Module 3 as the preferred routine recommendation. Module 1 combined multiple institutional control components and produced the greatest reduction in pest pressure and the largest yield gain, but it is best justified as a contingency module for heavy infestation or outbreak conditions. Module 3, which integrates plant oils, entomopathogenic fungi, and a selective insecticide, consistently resulted in substantial reduction of pest populations, demonstrating that effective control can be achieved with comparatively lower reliance on conventional chemical inputs. The slightly lower efficacy of Module 3, compared to Module 1, may be attributed to the absence of additional biological components such as parasitoids and scavenging beetles. However, its strong performance highlights the potential of combining plant oils and microbial agents to provide sustained pest suppression while minimizing the ecological risks associated with intensive insecticide use. Module 3 offers a more environmentally compatible and potentially farmer-friendly alternative, particularly in systems where conservation of natural enemies and reduction of chemical residues are prioritized. Thus, while Module 1 may be preferred for achieving maximum immediate control under high infestation pressure, Module 3 represents a balanced and sustainable IPM strategy for long-term management of *A. rugioperculatus*. These findings are consistent with earlier studies emphasizing the benefits of integrating plant oils, entomopathogenic fungi, and selective insecticides for whitefly management (19, 25).

The comparative analysis across treatments demonstrates that reliance on a single tactic is insufficient to curb RSW infestations, given the pest’s high reproductive potential and ability to exploit favorable conditions. Instead, the integrated approach evaluated here leverages the complementary strengths of plant oils, EPF, and insecticides. Plant oils such as castor and neem oils disrupt pest physiology and oviposition, while EPF like *L. lecanii* provide long-lasting microbial control that spreads epizootically within populations. Chemical insecticides, particularly acetamiprid, ensure rapid knockdown when pest populations exceed threshold levels. Such integration not only enhances overall efficacy but also reduces the frequency of chemical applications, lowering risks of resistance development and environmental contamination. A limitation of the present study is that the impact of treatments on natural enemies was not assessed. While Plant oils and entomopathogenic fungi are generally considered selective, insecticides such as acetamiprid may affect non-target organisms. Future studies should evaluate the compatibility of these IPM modules with natural enemies to ensure sustainable pest management.

Collectively, the results validate Module 3 as a robust integrated pest management (IPM) strategy against RSW in coconut ecosystems. Its consistent performance across two seasons highlights its reliability and scalability for wider adoption. Moreover, the findings align with national IPM programs that advocate blending biological control with selective chemical use to promote sustainable crop protection (25). Thus, the current study underscores the potential of multi-tactic IPM modules that harmonize efficacy, ecological safety, and farmer adoption feasibility for long-term management of *A. rugioperculatus*. From an environmental perspective, the integration of plant oils and EPF is generally considered more compatible with non-target organisms compared to sole reliance on chemical insecticides. However, these assertions are based on existing literature and indirect inference, as the present study did not quantify non-target effects. Therefore, future studies should include empirical evaluation of ecological safety under field conditions.

## 5 Conclusion

The study demonstrates that development of IPM modules for RSW should be judged on both efficacy and sustainability rather than on laboratory toxicity alone. Module 1 provided the greatest overall suppression and yield gain and may therefore be used as a benchmark or as an outbreak-management option under severe infestation. For routine field adoption, however, Module 3 is the more defensible recommendation because it integrates laboratory-validated botanical, microbial and need-based chemical tactics within a reduced-chemical framework. The manuscript should therefore conclude by recommending Module 3 as the preferred IPM option for long-term management of *A. rugioperculatus* in coconut under north-eastern Indian conditions, while positioning Module 1 as a high-suppression contingency module rather than the primary recommendation.

## Data Availability

The original contributions presented in the study are included in the article/**Supplementary Material**. Further inquiries can be directed to the corresponding author.
