# Supplementary material for: Integrated evaluation of plant oils, entomopathogenic fungi and insecticides against rugose spiralling whitefly (Aleurodicus rugioperculatus Martin) on coconut
Source: Front Insect Sci. 2026 Jun 9;6:1829366. doi: 10.3389/finsc.2026.1829366 (PMC13287131; doi:10.3389/finsc.2026.1829366)
Supplement: Supplementary file 1 [file Table1.docx]

**Supplementary files**

| **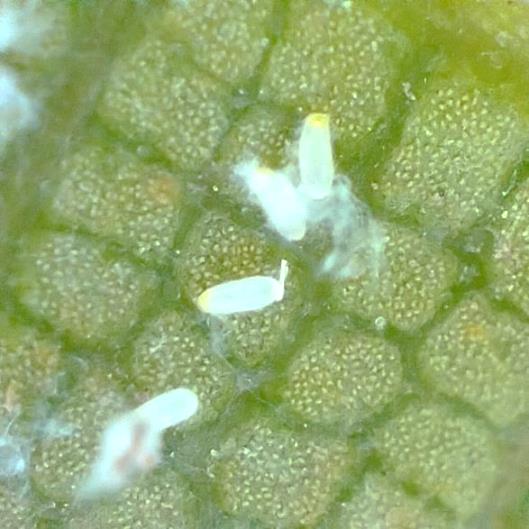** | **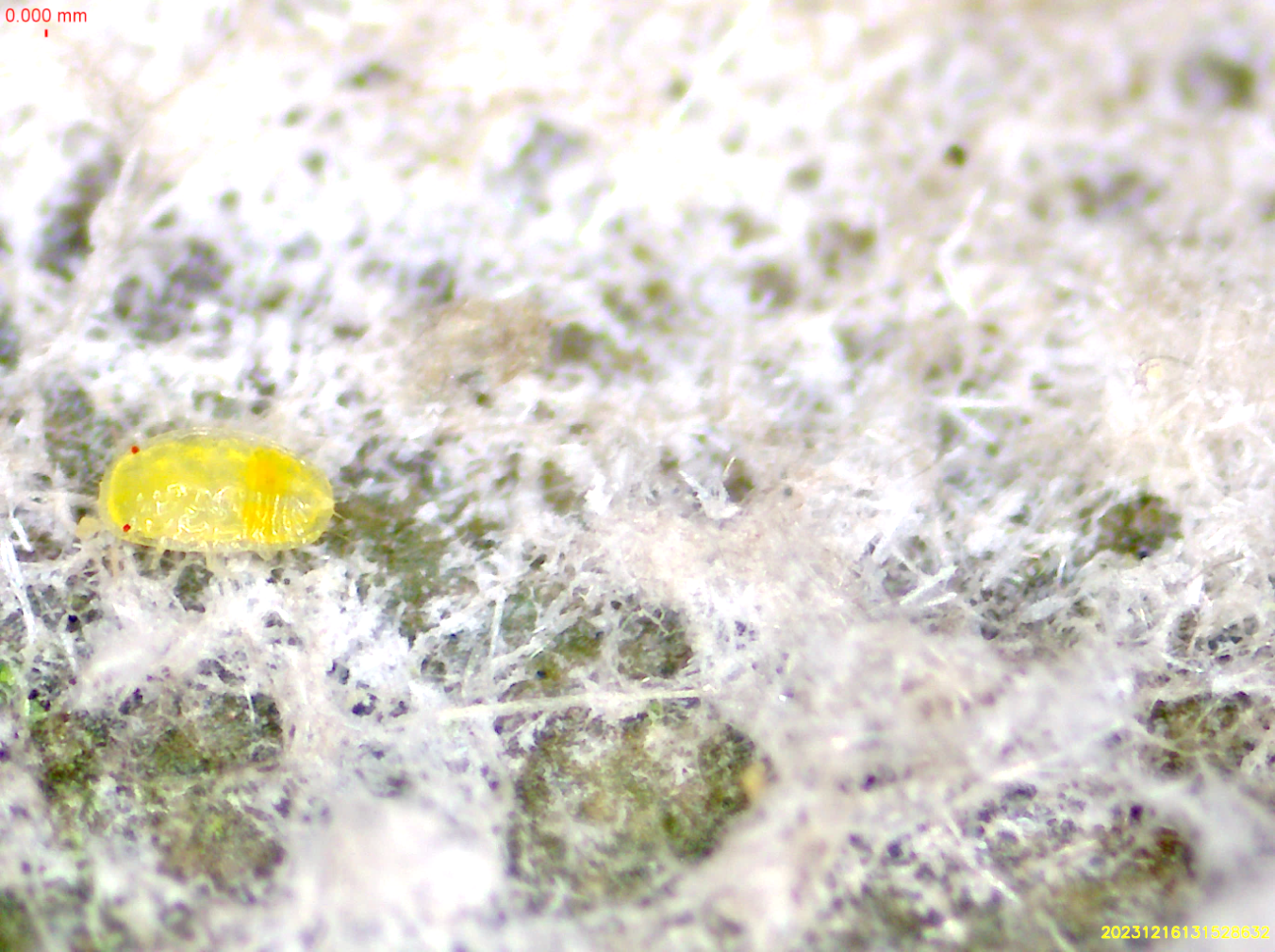** |
| --- | --- |
| **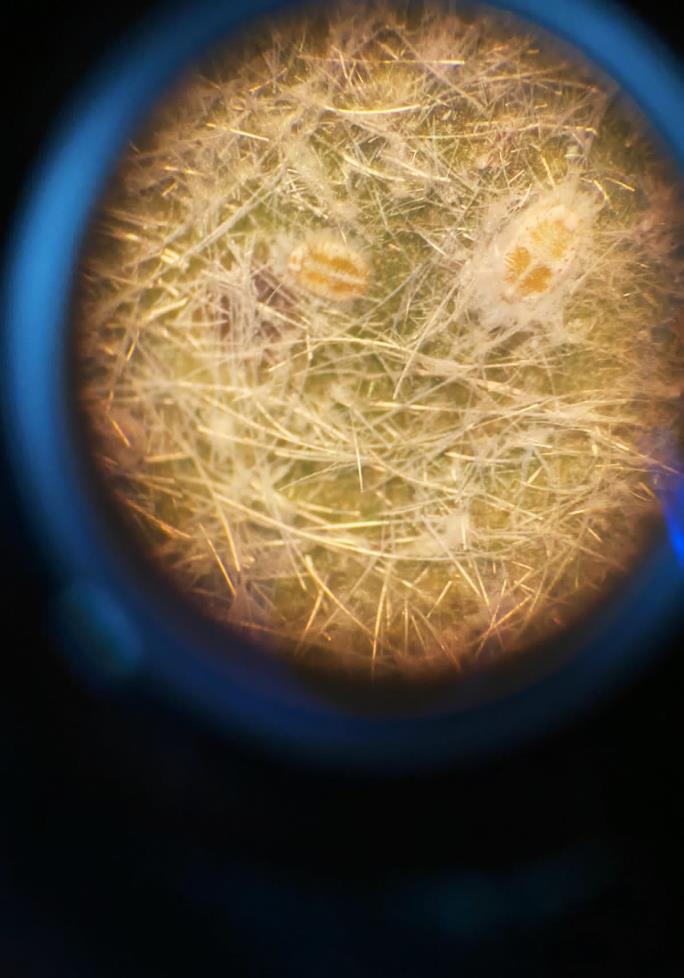** | **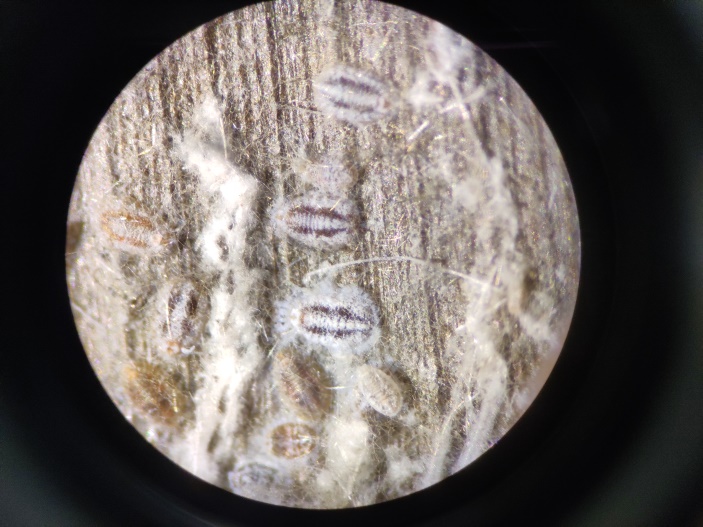** |
| **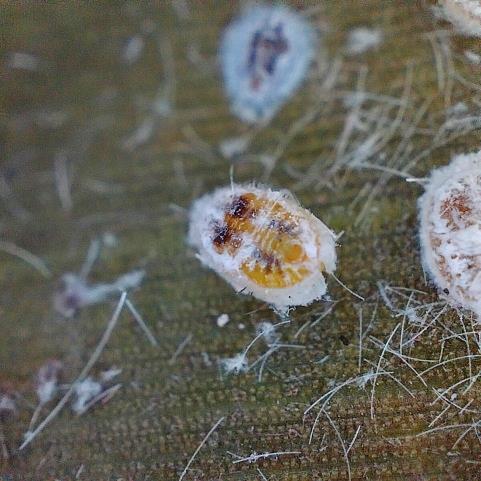** | **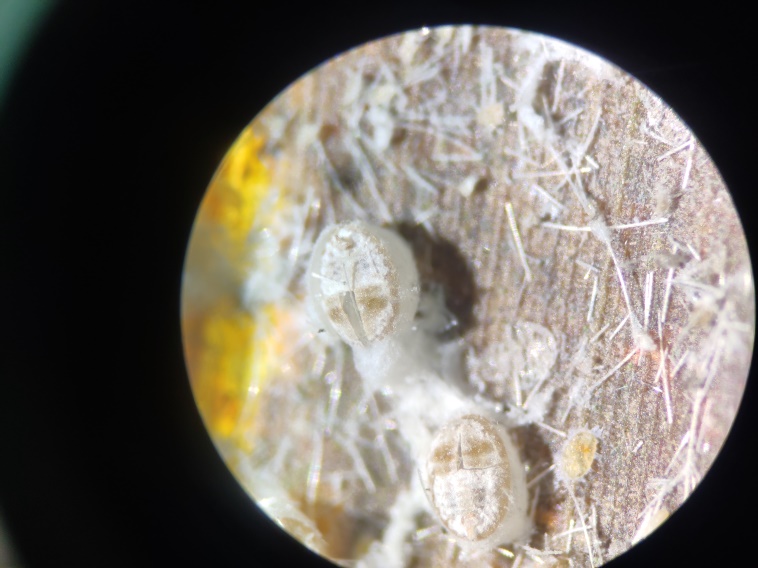** |

Supplementary figure 1: Different life stages of Rugose Spirialling Whitefly (a) Egg; (b) I instar (crawler); (c) II instar; (d) III instar; (e) IV instar (pseudo pupa); (f) Pupae with T shaped slit

**Supplementary Table 1: Effect of botanicals on mortality of *A. rugioperculatus* (adults) at different exposure period**

| **Concentration (%)** | **Mortality %** | | | | | | | | | | | |
| --- | --- | --- | --- | --- | --- | --- | --- | --- | --- | --- | --- | --- |
|  | **Jathropa** | | | **Karanj** | | | **Castor** | | | **Neem** | | |
|  | **24 HAT** | **48 HAT** | **72 HAT** | **24 HAT** | **48 HAT** | **72 HAT** | **24 HAT** | **48 HAT** | **72 HAT** | **24 HAT** | **48 HAT** | **72 HAT** |
| 3 | 35.00  (36.22) | 40.00  (39.19) | 53.33  (46.89) | 46.66  (43.07) | 51.66  (45.93) | 58.33  (49.78) | 66.66  (54.76) | 66.66  (54.76) | 70.00  (56.81) | 48.33  (44.02) | 58.33  (49.78) | 65.00  (53.74) |
| 2 | 31.66  (34.21) | 36.66  (37.24) | 48.33  (44.02) | 43.33  (41.14) | 48.33  (44.02) | 53.33  (46.89) | 58.33  (49.78) | 58.33  (49.78) | 61.66  (51.73) | 48.33  (44.02) | 50.00  (44.98) | 55.00  (47.86) |
| 1 | 26.66  (31.05) | 31.66  (34.21) | 41.66  (40.18) | 38.33  (38.22) | 43.33  (41.14) | 48.33  (44.02) | 50.00  (44.98) | 50.00  (44.98) | 53.33  (46.89) | 41.66  (40.18) | 45.00  (42.11) | 48.33  (44.02) |
| 0.5 | 21.66  (27.69) | 28.33  (32.12) | 36.66  (37.24) | 35.00  (36.25) | 38.33  (38.22) | 43.33  (41.14) | 40.00  (39.19) | 40.00  (39.19) | 43.33  (41.14) | 38.33  (38.22) | 40.00  (39.21) | 41.66  (40.18) |
| 0.2 | 18.33  (25.29) | 23.33  (28.84) | 28.33  (32.12) | 31.66  (34.21) | 33.33  (35.23) | 38.33  (38.22) | 33.33  (35.20) | 33.33  (35.20) | 35.00  (36.22) | 33.33  (35.23) | 38.33  (38.22) | 41.66  (40.18) |
| Control | 0.00  (0.33) | 0.00  (0.33) | 0.00  (0.33) | 0.00  (0.33) | 0.00  (0.33) | 0.00  (0.33) | 0.00  (0.33) | 0.00  (0.33) | 0.00  (0.33) | 0.00  (0.33) | 0.00  (0.33) | 0.00  (0.33) |
| S.Ed (±) | 2.54 | 2.54 | 2.15 | 1.92 | 2.15 | 2.15 | 3.33 | 3.33 | 2.88 | 3.46 | 3.46 | 3.60 |
| CD (P=0.05) | 5.60 | 5.60 | 4.74 | 4.24 | 4.74 | 4.74 | 7.34 | 7.34 | 6.35 | 7.64 | 7.64 | 7.93 |

*HAT- Hours After Treatment, data are mean of 3 replications, figures in parentheses are angular transformed values*

**Supplementary Table 2: Effect of botanicals on mortality of *A. rugioperculatus* (later instar nymphs) at different exposure period**

| **Concentration (%)** | **Mortality %** | | | | | | | | | | | |
| --- | --- | --- | --- | --- | --- | --- | --- | --- | --- | --- | --- | --- |
|  | **Jathropa** | | | **Karanj** | | | **Castor** | | | **Neem** | | |
|  | **24 HAT** | **48 HAT** | **72 HAT** | **24 HAT** | **48 HAT** | **72 HAT** | **24 HAT** | **48 HAT** | **72 HAT** | **24 HAT** | **48 HAT** | **72 HAT** |
| 3 | 31.66  (34.13) | 36.66  (37.19) | 50.00  (44.98) | 43.33  (41.13) | 48.33  (44.02) | 55.00  (47.86) | 60.00  (50.74) | 63.33  (52.77) | 66.66  (54.81) | 45.00  (42.10) | 55.00  (47.86) | 61.66  (51.75) |
| 2 | 28.33  (32.07) | 33.33  (35.20) | 45.00  (42.11) | 40.00  (39.19) | 45.00  (42.10) | 50.00  (44.98) | 51.66  (45.93) | 55.00  (47.86) | 58.33  (49.80) | 45.00  (42.10) | 50.00  (44.98) | 56.66  (48.82) |
| 1 | 23.33  (28.76) | 28.33  (32.07) | 38.33  (38.22) | 35.00  (36.22) | 40.00  (39.19) | 45.00  (42.10) | 43.33  (41.14) | 46.66  (43.07) | 50.00  (44.98) | 40.00  (39.19) | 45.00  (42.10) | 50.00  (44.98) |
| 0.5 | 18.33  (25.18) | 25.00  (29.91) | 33.33  (35.23) | 31.66  (34.21) | 35.00  (36.22) | 40.00  (39.19) | 33.33  (35.10) | 36.66  (37.18) | 40.00  (39.19) | 36.66  (37.24) | 41.66  (40.18) | 46.66  (43.07) |
| 0.2 | 15.00  (22.58) | 20.00  (26.44) | 25.00  (29.91) | 28.33  (32.12) | 30.00  (33.14) | 35.00  (36.22) | 23.33  (28.52) | 30.00  (33.06) | 31.66  (34.13) | 30.00  (33.14) | 35.00  (36.22) | 40.00  (39.19) |
| Control | 0.00  (0.33) | 0.00  (0.33) | 0.00  (0.33) | 0.00  (0.33) | 0.00  (0.33) | 0.00  (0.33) | 0.00  (0.330 | 0.00  (0.33) | 0.00  (0.33) | 0.00  (0.33) | 0.00  (0.33) | 0.00  (0.33) |
| S.Ed (±) | 4.51 | 4.41 | 2.15 | 3.33 | 3.84 | 3.72 | 5.35 | 5.00 | 4.41 | 3.46 | 3.46 | 3.72 |
| CD (P=0.05) | 9.94 | 9.71 | 4.74 | 7.34 | 8.47 | 8.21 | 11.80 | 11.01 | 9.71 | 7.64 | 7.64 | 8.21 |

*HAT- Hours After Treatment, data are mean of 3 replications, figures in parentheses are angular transformed values*

**Supplementary Table 3: Effect of entomopathogenic fungi on mortality of *A. rugioperculatus* (adults) at different exposure period**

| **Spore dilution**  **(cfu/ml)** | **Mortality %** | | | | | | | | | | | |
| --- | --- | --- | --- | --- | --- | --- | --- | --- | --- | --- | --- | --- |
|  | *Beauveria bassiana* | | | ***Metarhizium anisopliae*** | | | ***L. lecanii*** | | | ***I. fumosorosea*** | | |
|  | **3 DAT** | **5 DAT** | **7 DAT** | **3 DAT** | **5 DAT** | **7 DAT** | **3 DAT** | **5 DAT** | **7 DAT** | **3 DAT** | **5 DAT** | **7 DAT** |
| 10^3^ | 33.33  (35.27) | 46.66  (43.07) | 48.33  (44.02) | 6.66  (14.75) | 8.33  (16.59) | 10.00  (18.42) | 31.66  (34.21) | 38.33  (38.22) | 41.66  (40.18) | 41.66  (40.18) | 43.33  (41.14) | 53.33  (46.89) |
| 10^5^ | 45.00  (42.10) | 50.00  (44.98) | 51.66  (45.93) | 8.33  (16.59) | 10.00  (18.42) | 11.66  (19.87) | 46.66  (43.07) | 55.00  (47.86) | 58.33  (49.81) | 60.00  (50.76) | 61.66  (51.73) | 65.00  (53.74) |
| 10^7^ | 53.33  (46.89) | 58.33  (49.78) | 61.66  (51.73) | 23.33  (28.84) | 26.66  (31.05) | 28.33  (32.07) | 53.33  (46.89) | 70.00  (56.81) | 75.00  (60.05) | 66.66  (54.72) | 78.33  (62.26) | 85.00  (67.37) |
| 10^8^ | 48.33  (44.02) | 53.33  (46.89) | 56.66  (48.81) | 10.00  (18.04) | 11.66  (19.87) | 13.33  (21,32) | 40.00  (39.19) | 60.00  (50.76) | 65.00  (53.74) | 63.33  (52.72) | 65.00  (53.74) | 71.66  (57.83) |
| 10^9^ | 33.33  (35.27) | 38.33  (38.22) | 41.66  (40.18) | 3.33  (8.61) | 5.00  (12.91) | 6.66  (14.75) | 38.33  (38.22) | 36.66  (37.24) | 40.00  (39.21) | 46.66  (43.07) | 51.66  (45.93) | 60.00  (50.76 |
| Control | 0.00  (0.33) | 0.00  (0.33) | 0.00  (0.33) | 0.00  (0.33) | 0.00  (0.33) | 0.00  (0.33) | 0.00  (0.33) | 0.00  (0.33) | 0.00  (0.33) | 0.00  (0.33) | 0.00  (0.33) | 0.00  (0.33) |
| S.Ed (±) | 2.54 | 1.92 | 2.15 | 2.54 | 1.66 | 2.54 | 2.54 | 3.19 | 3.60 | 2.54 | 2.54 | 3.19 |
| CD | 5.60 | 4.24 | 4.74 | 5.60 | 3.67 | 5.60 | 5.60 | 7.03 | 7.93 | 5.60 | 5.60 | 7.03 |

*DAT- Days After Treatment, data are mean of 3 replications, figures in parentheses are angular transformed values*

**Supplementary Table 4: Effect of entomopathogenic fungi on mortality of *A. rugioperculatus* (nymphs) at different exposure period**

| **Spore dilution**  **(cfu/ml)** | **Mortality %** | | | | | | | | | | | |
| --- | --- | --- | --- | --- | --- | --- | --- | --- | --- | --- | --- | --- |
|  | *Beauveria bassiana* | | | ***Metarhizium anisopliae*** | | | ***L. lecanii*** | | | ***I. fumosorosea*** | | |
|  | **3 DAT** | **5 DAT** | **7 DAT** | **3 DAT** | **5 DAT** | **7 DAT** | **3 DAT** | **5 DAT** | **7 DAT** | **3 DAT** | **5 DAT** | **7 DAT** |
| 10^3^ | 30.00  (33.14) | 43.33  (41.14) | 45.00  (42.10) | 3.33  (8.61) | 5.00  (10.44) | 6.66  (14.75) | 28.33  (32.12) | 35.00  (36.25) | 38.33  (38.22) | 38.33  (38.22) | 40.00  (39.19) | 50.00  (44.98) |
| 10^5^ | 41.66  (40.18) | 46.66  (43.07) | 48.33  (44.02) | 5.00  (12.91) | 6.66  (14.75) | 8.33  (16.59) | 43.33  (41.13) | 51.66  (45.93) | 55.00  (47.86) | 56.66  (48.82) | 58.33  (49.78) | 61.66  (51.75) |
| 10^7^ | 50.00  (44.98) | 55.00  (47.86) | 58.33  (49.78) | 20.00  (26.44) | 23.33  (28.84) | 25.00  (29.91) | 50.00  (44.98) | 66.66  (54.72) | 71.66  (57.83) | 63.33  (52.72) | 75.00  (59.97) | 81.66  (64.66) |
| 10^8^ | 45.00  (42.10) | 50.00  (44.98) | 53.33  (46.90) | 6.66  (12.28) | 8.33  (16.59) | 10.00  (18.04) | 36.66  (37.18) | 56.66  (48.81) | 61.66  (51.73) | 60.00  (50.74) | 61.66  (51.75) | 68.33  (55.74) |
| 10^9^ | 30.00  (33.14) | 35.00  (36.22) | 38.33  (38.22) | 1.66  (4.30) | 1.66  (4.30) | 3.33  (8.61) | 35.00  (36.22) | 33.33  (35.20) | 36.66  (37.24) | 43.33  (41.13) | 48.33  (44.02) | 56.66  48.81) |
| Control | 0.00  (0.33) | 0.00  (0.33) | 0.00  (0.33) | 0.00  (0.33) | 0.00  (0.33) | 0.00  (0.33) | 0.00  (0.33) | 0.00  (0.33) | 0.00  (0.33) | 0.00  (0.33) | 0.00  (0.33) | 0.00  (0.33) |
| S.Ed (±) | 3.46 | 3.19 | 3.04 | 2.88 | 2.54 | 2.88 | 4.08 | 2.54 | 2.54 | 3.04 | 2.88 | 1.56 |
| CD | 7.64 | 7.03 | 6.70 | 6.35 | 5.60 | 6.35 | 8.99 | 5.60 | 5.60 | 6.70 | 6.35 | 3.45 |

*DAT- Days After Treatment, data are mean of 3 replications, figures in parentheses are angular transformed values*

**Supplementary Table 5: Effect of insecticides on mortality of *A. rugioperculatus* (adults) at different exposure period**

| **Mortality %** | | | | | | | | | | | | | | | |
| --- | --- | --- | --- | --- | --- | --- | --- | --- | --- | --- | --- | --- | --- | --- | --- |
| **Concentration (%)** | Thiamethoxam 25% WG | | | **Concentration (%)** | Spiromesifen 22.9% SC | | | **Concentration (%)** | Acetamiprid 20% SP | | | **Concentration (%)** | Imidacloprid 17.8% SL | | |
|  | **24 HAT** | **48 HAT** | **72 HAT** |  | **24 HAT** | **48 HAT** | **72 HAT** |  | **24 HAT** | **48 HAT** | **72 HAT** |  | **24 HAT** | **48 HAT** | **72 HAT** |
| 1 | 60.00  (50.76) | 61.66  (51.73) | 63.33  (52.72) | 1 | 53.33  (46.89) | 55.00  (47.86) | 55.00  (47.86) | 1 | 80.00  (63.40) | 83.33  (65.92) | 85.00  (67.18) | 0.05 | 65.00  (53.74) | 66.66  (54.81) | 68.33  (55.79) |
| 0.5 | 46.66  (43.07) | 48.33  (44.02) | 50.00  (44.98) | 0.9 | 46.66  (43.07) | 48.33  (44.02) | 48.33  (44.02) | 0.5 | 68.33  (55.79) | 68.33  (55.74) | 71.66  (57.83) | 0.01 | 55.00  (47.86) | 56.66  (48.84) | 58.33  (49.80) |
| 0.2 | 38.33  (38.22) | 40.00  (39.21) | 41.66  (40.18) | 0.8 | 40.00  (39.21) | 41.66  (40.18) | 41.66  (40.18) | 0.2 | 60.00  (50.76) | 61.66  (51.73) | 65.00  (53.70) | 0.005 | 45.00  (42.10) | 45.00  (42.10) | 46.66  (43.06) |
| 0.1 | 26.66  (31.05) | 28.33  (32.12) | 31.66  (34.21) | 0.5 | 30.00  (33.19) | 28.33  (32.12) | 31.66  (34.21) | 0.1 | 48.33  (44.02) | 50.00  (44.98) | 51.66  (45.93) | 0.003 | 35.00  (36.22) | 35.00  (36.22) | 36.66  (37.18) |
| 0.05 | 18.33  (25.29) | 20.00  (26.55) | 21.66  (27.69) | 0.1 | 20.00  (26.55) | 21.66  (27.69) | 21.66  (27.69) | 0.05 | 38.33  (38.20) | 40.00  (39.19) | 41.66  (40.16) | 0.001 | 25.00  (29.67) | 25.00  (29.67) | 26.66  (30.93) |
| Control | 0.00  (0.33) | 0.00  (0.33) | 0.00  (0.33) | Control | 0.00  (0.33) | 0.00  (0.33) | 0.00  (0.33) | Control | 0.00  (0.33) | 0.00  (0.33) | 0  (0.33) | Control | 0.00  (0.33) | 0.00  (0.33) | 0.00  (0.33) |
| S.Ed (±) | 2.54 | 2.35 | 2.54 | S.Ed (±) | 1.36 | 2.54 | 2.54 | S.Ed (±) | 3.72 | 2.88 | 1.66 | S.Ed (±) | 4.71 | 5.44 | 5.18 |
| CD | 5.60 | 5.19 | 5.60 | CD | 2.99 | 5.60 | 5.60 | CD | 8.21 | 6.35 | 5.19 | CD | 10.38 | 11.99 | 11.41 |

*HAT- Hours After Treatment, data are mean of 3 replications, figures in parentheses are angular transformed values, CD* (P=0.05)

**Supplementary Table 6: Effect of insecticides on mortality of *A. rugioperculatus* (later instar nymphs) at different exposure period**

| **Mortality %** | | | | | | | | | | | | | | | |
| --- | --- | --- | --- | --- | --- | --- | --- | --- | --- | --- | --- | --- | --- | --- | --- |
| **Concentration (%)** | Thiamethoxam 25% WG | | | **Concentration (%)** | Spiromesifen 22.9% SC | | | **Concentration (%)** | Acetamiprid 20% SP | | | **Concentration (%)** | Imidacloprid 17.8% SL | | |
|  | **24 HAT** | **48 HAT** | **72 HAT** |  | **24 HAT** | **48 HAT** | **72 HAT** |  | **24 HAT** | **48 HAT** | **72 HAT** |  | **24 HAT** | **48 HAT** | **72 HAT** |
| 1 | 63.33  (52.72) | 65.00  (53.70) | 66.66  (54.72) | 1 | 56.66  (48.81) | 58.33  (49.78) | 58.33  (49.78) | 1 | 83.33  (65.92) | 86.66  (68.63) | 88.33  (70.08) | 0.05 | 68.33  (55.74) | 70.00  (56.81) | 71.66  (57.83) |
| 0.5 | 50.00  (44.98) | 51.66  (45.93) | 53.33  (46.89) | 0.9 | 50.00  (44.98) | 51.66  (45.93) | 51.66  (45.93) | 0.5 | 71.66  (57.83) | 71.66  (57.83) | 73.33  (58.90) | 0.01 | 58.33  (49.78) | 60.00  (50.76) | 61.66  (51.73) |
| 0.2 | 41.66  (40.18) | 43.33  (41.14) | 45.00  (42.10) | 0.8 | 43.33  (41.14) | 45.00  (42.10) | 45.00  (42.10) | 0.2 | 63.33  (52.72) | 65.00  (53.70) | 66.66  (54.72) | 0.005 | 48.33  (44.02) | 48.33  (44.02) | 50.00  (44.98) |
| 0.1 | 30.00  (33.19) | 31.66  (34.21) | 35.00  (36.22) | 0.5 | 33.33  (35.23) | 31.66  (34.21) | 35.00  (36.22) | 0.1 | 51.66  (45.93) | 53.33  (46.89) | 53.33  (46.89) | 0.003 | 38.33  (38.22) | 38.33  (38.22) | 40.00  (39.19) |
| 0.05 | 21.66  (27.69) | 23.33  (28.84) | 25.00  (29.91) | 0.1 | 23.33  (28.84) | 25.00  (29.91) | 25.00  (29.91) | 0.05 | 41.66  (40.18) | 43.33  (41.14) | 43.33  (41.14) | 0.001 | 28.33  (32.00) | 28.33  (32.00) | 30.00  (33.14) |
| Control | 0.00  (0.33) | 0.00  (0.33) | 0.00  (0.33) | Control | 0.00  (0.33) | 0.00  (0.33) | 0.00  (0.33) | Control | 0.00  (0.33) | 0.00  (0.33) | 0.00  (0.33) | Control | 0  (0.33) | 0  (0.33) | 0  (0.33) |
| S.Ed (±) | 1.66 | 2.15 | 2.88 | S.Ed (±) | 1.92 | 2.88 | 3.19 | S.Ed (±) | 2.15 | 2.15 | 2.15 | S.Ed (±) | 3.19 | 3.72 | 3.19 |
| CD | 3.67 | 4.74 | 6.35 | CD | 4.24 | 6.35 | 7.03 | CD | 4.74 | 4.74 | 4.74 | CD | 7.03 | 8.21 | 7.03 |

*HAT- Hours After Treatment, data are mean of 3 replications, figures in parentheses are angular transformed values, CD* (P=0.05)

**Table 6: Effect of different IPM modules on *A. rugioperculatus* incidence and yield (pooled over two seasons)**

| **Module** | **Adult reduction (%)** | **Nymph reduction (%)** | **Mean yield (t/ha)** | **Yield increase over control (%)** | **Benefit:cost ratio** | **Rank** |
| --- | --- | --- | --- | --- | --- | --- |
| Module 1 | 73.5 ± 2.1 | 72.0 ± 2.0 | 2.85 ± 0.08 | 28.4 | 1:4.6 | *I* |
| Module 2 | 64.0 ± 2.5 | 62.3 ± 2.2 | 2.68 ± 0.06 | 23.9 | 1:4.1 | *II* |
| Module 3 | 42.1 ± 2.8 | 40.6 ± 2.6 | 2.35 ± 0.07 | 15.6 | 1:3.2 | *III* |
| Module 4 | 37.8 ± 2.9 | 35.4 ± 2.7 | 2.26 ± 0.05 | 13.2 | 1:2.9 | *IV* |
